# Prediction of long-term survival after gastrectomy using random survival forests

**DOI:** 10.1093/bjs/znab237

**Published:** 2021-07-16

**Authors:** S A Rahman, N Maynard, N Trudgill, T Crosby, M Park, H Wahedally, T J Underwood, D A Cromwell, the NOGCA Project Team and AUGIS

**Affiliations:** 1 School of Cancer Sciences, Faculty of Medicine, University of Southampton, Southampton, UK; 2 Clinical Effectiveness Unit, Royal College of Surgeons of England, London, UK; 3 Oxford University Hospitals NHS Trust, Oxford, UK; 4 Sandwell and West Birmingham NHS Trust, Birmingham, UK; 5 Velindre Cancer Centre, Cardiff, UK

## Abstract

**Background:**

No well validated and contemporaneous tools for personalized prognostication of gastric adenocarcinoma exist. This study aimed to derive and validate a prognostic model for overall survival after surgery for gastric adenocarcinoma using a large national dataset.

**Methods:**

National audit data from England and Wales were used to identify patients who underwent a potentially curative gastrectomy for adenocarcinoma of the stomach. A total of 2931 patients were included and 29 clinical and pathological variables were considered for their impact on survival. A non-linear random survival forest methodology was then trained and validated internally using bootstrapping with calibration and discrimination (time-dependent area under the receiver operator curve (tAUC)) assessed.

**Results:**

The median survival of the cohort was 69 months, with a 5-year survival of 53.2 per cent. Ten variables were found to influence survival significantly and were included in the final model, with the most important being lymph node positivity, pT stage and achieving an R0 resection. Patient characteristics including ASA grade and age were also influential. On validation the model achieved excellent performance with a 5-year tAUC of 0.80 (95 per cent c.i. 0.78 to 0.82) and good agreement between observed and predicted survival probabilities. A wide spread of predictions for 3-year (14.8–98.3 (i.q.r. 43.2–84.4) per cent) and 5-year (9.4–96.1 (i.q.r. 31.7–73.8) per cent) survival were seen.

**Conclusions:**

A prognostic model for survival after a potentially curative resection for gastric adenocarcinoma was derived and exhibited excellent discrimination and calibration of predictions.

## Teaser

This article describes the derivation and validation of a population-based machine learning model to predict overall survival after surgery for gastric adenocarcinoma. It provided excellent discrimination and calibration and will be of use to all clinicians treating patients with this disease.

**Highlights**

- No well validated contemporaneous prognostic model for gastric adenocarcinoma is in widespread clinical use
- This study describes the derivation of a random survival forest model using routine data from a large population dataset
- The model performed well on internal validation with a time-dependent area under the receiver operator curve of 0.80 and excellent calibration
- A wide range of predictions were yielded for each TNM stage
- After appropriate external validation, it could provide utility in both prognostication for patients and for benchmarking of treatment responses.

## Introduction

Gastric cancer is among the most common causes of cancer and cancer mortality worldwide, with an estimated 1 000 000 cases and 783 000 deaths in 2018^1^. Similar to oesophageal cancer, gastric cancer is more common among men than women, and the majority of cases occur in East Asia, where an incidence of up to 32 per 100 000 is seen overall. In comparison, in Northern Europe, the incidence is 6.2 per 100 000 in men and 3.1 per 100 000 in women. In England and Wales there is a significant burden of disease, with 5972 cases of gastric adenocarcinoma diagnosed between April 2017 and March 2019^2^, and among those only around one third suitable for curative treatment at presentation.

Among western populations, stratification of patient outcomes is limited to TNM stage, with a lack of tools for personalized prognostication which incorporate other variables known to influence survival. In a recent systematic review of prognostic tools in oesophageal and gastric cancer^3^ only one model suitable for gastric cancer was considered to be methodologically sound^4^, however this study was conducted in 2003 before the widespread use of neoadjuvant treatment and was limited to patients undergoing R0 resection. A further review^5^ reached similar conclusions, identifying generally poor methodology and poor validation strategies among studies. Accurate postoperative prognostication is important as it allows personalized planning of both follow-up and potential adjuvant treatment in addition to accurate comparison of different treatment regimens between groups of patients. No such tool to achieve this exists to date.

It is likely that in the future in-depth analysis of patients’ cancers will allow for a high level of accuracy of prognostication both in the pre- and post-treatment settings, however these methodologies are not yet widely available, are time consuming and expensive. Optimal use of clinical data is therefore key. Machine learning techniques which incorporate non-linear effects, interactions between variables and time-varying effects have the potential to capture additional information from routine clinical data that may be missed by traditional prognostic models such as the Cox proportional hazards.

Recently, data from the England and Wales National Oeosophago-Gastric Cancer Audit (NOGCA) has been used to derive a random survival forest (RSF)^6^ model for prognosis after oesophagectomy with considerable accuracy in excess of a Cox proportional hazards model^7^. This study aims to apply a similar methodology to patients diagnosed with gastric adenocarcinoma in England and Wales from 2012–2018 with the goal of deriving an accurate prediction tool for overall survival after surgery.

## Methods

This study used a dataset of cases identified from the NOGCA as has been described previously^7^. Data entry into the NOGCA has been compulsory for all patients diagnosed with epithelial cancer of the stomach or oesophagus since 2012, with named clinicians responsible for its collection as part of the multidisciplinary team. Each year, centres and surgeons are sent their results prior to publication and are asked to update incomplete or inaccurate data. Case ascertainment is evaluated using the national administrative hospital databases (Hospital Episode Statistics in England and its Welsh equivalent), and is estimated to exceed 99 per cent for patients who undergo curative surgery. The dataset used for this study included patients diagnosed between April 2012 and March 2018^8^. Details of neoadjuvant and adjuvant treatment were cross-referenced with the Systemic Anti-Cancer Therapy (SACT) dataset. A total of 4238 patients who underwent a gastrectomy for adenocarcinoma of the stomach or gastro-oesophageal junction (Siewert III) were identified. Exclusion criteria included overt metastatic disease at resection (pM1), death prior to discharge from hospital or if fewer than 15 lymph nodes were examined from the resection specimen (suggesting the patient may have been incompletely staged)^9^. A comparison of these patients with the main study cohort is provided in *Table S1* and *Fig. S1*. A complete list and details of exclusions to reach the final sample size of 2931 cases is given in *Fig. S2*. The primary outcome was defined as overall survival from time of hospital discharge, with survival confirmed using the Office for National Statistics death register.

Variables collected in the audit were considered for inclusion if there was a plausible relationship with survival, completeness in excess of 50 per cent and a frequency of at least 1 per cent in the cohort. For this study a total of 29 variables were identified as potential predictors (*Table S2*), including patient characteristics, preoperative tumour staging, complications of surgery, postoperative pathology and neoadjuvant/adjuvant treatment. Type of operation (for example, distal gastrectomy, total gastrectomy, extended total gastrectomy) was considered but omitted as it was almost exclusively correlated with site of tumour. Anastomotic leak was defined as severe disruption to the anastomosis (detected clinically or radiologically) including those patients managed actively and conservatively. An R0 resection was defined as complete macro-/microscopic resection of tumour with negative longitudinal and circumferential resection margins. The authors considered unit volume as combination of major upper gastrointestinal resections per year (major gastrectomy and oesophagectomy) as per published research^10^ and in line with NHS commissioning guidelines^11^ and also separately for gastrectomy alone.

TNM staging was conducted using the 8th edition staging manual^12^. There was at least one data point missing in 671 cases (22.9 per cent). The most frequently missing characteristics were return to theatre (15.1 per cent), cT stage (12.4 per cent) and differentiation grade (6.5 per cent). All other variables had less than 5 per cent missing data. Missing data were assumed to be missing at random and handled using multiple imputation by chained equations^13^ with 10 imputed datasets.

In order to produce a more concise model with increased generalizability, a variable selection step was conducted using the Boruta method^14^. Boruta identifies core variables by comparing the importance of candidate variables in a random forest to a corresponding set of ‘shadow’ variables, which are versions of each variable with their data randomized. Variables with importance significantly greater than all of the shadow variables are selected as important and retained, and variables with importance significantly less than the highest shadow variable are selected as unimportant and removed. This process is repeated with the decreasing number of uncertain variables until all are sorted into important or unimportant. It has been found to be more accurate than other approaches in variable selection in high-dimensional data^15^, particularly in large datasets^16^, and has been used in a variety of settings^17–19^. In this study variables were selected from complete cases only (2304 patients).

Identified important variables were then used to train an RSF using the Ranger^20^ package in R (R Foundation for Statistical Computing, Vienna, Austria). A random forest here is comprised of several hundred survival trees, each derived from different subpopulations of the cohort. Within each tree the binary split (for example, zero positive lymph nodes *versus* one or more positive lymph nodes) that gives the biggest difference in survival (as measured by the log rank test) is identified. The tree undergoes progressively more splits until a predetermined end point is reached. The random forest is then the mean output of all the decision trees. Parameters of the RSF that influence how it generates predictions, that is, number of trees, number of variables per tree and minimum node size, were selected to minimize out-of-sample error within the random forest. As multiple imputation was employed to address missing data, a means of pooling the outcomes from the imputed datasets is required. Here, as previously^7^, models were generated on each imputed dataset and predictions from each were combined after a log-log transformation^21^^,^^22^.

As the model incorporates both variable interaction and non-linear time effects, expressing the effect of individual variables is difficult. The hazard ratio is less appropriate as it assumes an exponential survival distribution and proportional hazards (that is, consistency of effect of variables over time)^23^. Use of the restricted mean survival time (RMST) has been proposed to address scenarios where the proportional hazards assumption does not hold true^24^, allowing for comparisons by absolute difference or ratio^25^ and is increasingly thought to be a more appropriate means of comparing survival outcomes and treatment effects^26–29^. Survival curves are first generated for each variable as the average predictions yielded for that variable. The RMST is then the area under each survival curve, the absolute difference in RMST between two factors (for example, R0 *versus* R1) is termed the life expectancy difference (LED) and the ratio between them the life expectancy ratio (LER). The LED and LER readily provide the absolute or relative gain/loss of life for each variable for the period of follow-up.

The internal validity of the model was quantified using 1000 replications of the bootstrap with replacement and the 0.632 estimator^30^. Discrimination was assessed using the time-dependent area under the receiver operator curve (tAUC)^31^, which corresponds to the proportion of random pairs of cases where one patient is alive and one dead at a specified time point where the model has correctly ordered their probability of survival having weighted for censoring. Calibration was assessed quantitatively using the integrated Brier score^32^^,^^33^, as a measure of overall error of predictions with a value closer to zero being better. Visual assessment of calibration was conducted by comparing predicted survival to observed (Kaplan–Meier) survival at specified time points. All analyses were conducted in R^34^, and the study was conducted to comply with the TRIPOD criteria^35^. Complete code to reproduce the analysis is available on request, and instructions for external validation are provided in the *Supplementary material*.

The study is exempt from UK National Research Ethics Committee approval as it involved secondary analysis of an existing dataset of anonymized data. The NOGCA has approval for processing health care information under Section 251 (reference number: ECC 1–06 (c)/2011) for all National Health Service (NHS) patients diagnosed with oesophagogastric cancer in England and Wales. Data for this study are based on patient-level information collected by the NHS, as part of the care and support of patients with cancer. Patient consent for publication was not required.

## Results

**Fig. 1:**
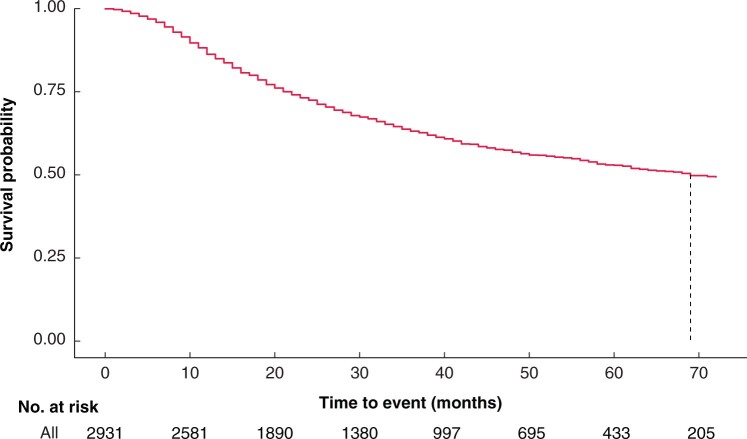
Kaplan–Meier survival of study cohort . The vertical dotted line represents the median survival, 69 months

The study population included 2931 patients who underwent a gastrectomy with a histologically proven diagnosis of adenocarcinoma. Patients were followed up for a median of 44 months, there were 1071 recorded deaths and the median survival was 69 months. At 3 and 5 years, survival was 63.5 and 53.2 per cent respectively (*Fig. 1*).

A median of 27 (range 15–109) lymph nodes were examined and at least one node contained tumour in the majority of patients (1635 of 2931, 55.8 per cent). Extent of nodal dissection was recorded as D2 in 2425 cases (82.7 per cent). Neoadjuvant chemotherapy was used in 48.0 per cent. Demographics of the population were as expected with 65.3 per cent males and a median age at diagnosis of 71 years. The vast majority of cases were undertaken in high-volume centres, with 91.6 per cent occurring in centres performing more than 30 major upper gastrointestinal resections per year. These characteristics are summarized in *Table 1*.

**Table 1:** Clinical and pathological characteristics of study cohort

Characteristic		Count	Survival at 5 years (%)	Characteristic		Count	Survival at 5 years (%)
**Age (years)**	18–50	263 (9.0)	64	Annual volume of major upper gastrointestinal resections*	1–30	245 (8.4)	50.5
	51–60	417 (14.2)	55.9		31–60	1563 (53.3)	52.5
	61–70	741 (25.3)	55.9		60+	1123 (38.3)	54.6
	71–80	1169 (39.9)	49.7	**Annual volume of major gastrectomy**	1–15	834 (28.1)	52.6
	>80	341 (11.6)	48.3		16–30	1654 (55.7)	51.2
**Gender**	Female	1017 (34.7)	55.8		30+	383 (12.9)	60.9
	Male	1914 (65.3)	51.7	**Surgical approach**	Laparoscopic	439 (15.0)	60.2
**Site of tumour**	Siewert III	416 (14.2)	41.1		Open	2492 (85.0)	52
	Fundus	195 (6.7)	56.2	**Surgical complication**	No	2240 (76.4)	53.9
	Body	1250 (42.6)	55.4		Yes	679 (23.2)	50.8
	Antrum	689 (23.5)	56.9		Missing	12 (0.4)	37.5
	Pylorus	381 (13.0)	50.7	**Anastomotic leak** ^†^	No	2826 (96.4)	53.5
**cT**	T0/is/1	295 (10.1)	79.9		Yes	93 (3.2)	45.4
	T2	579 (19.8)	59.1		Missing	12 (0.4)	37.5
	T3	1218 (41.6)	46	**pT/ypT stage**	T0	116 (4.0)	79.4
	T4	476 (16.2)	44.6		T1	591 (20.2)	81.2
	Missing	363 (12.4)	55.2		T2	454 (15.5)	66.7
**cN**	N0	1460 (49.8)	59.8		T3	1004 (34.3)	47.4
	N1	882 (30.1)	48.2		T4	766 (26.1)	27.5
	N2	369 (12.6)	44.7	**pN/ypN stage**	N0	1296 (44.2)	75.2
	N3	108 (3.7)	28.7		N1	495 (16.9)	55.4
	Missing	112 (3.8)	53.4		N2	513 (17.5)	38.6
**WHO performance status**	0	1409 (48.1)	56.6		N3	627 (21.4)	19.4
	1	1201 (41.0)	52.4	**R0 resection**	Yes	2663 (90.1)	56.6
	2	288 (9.8)	42.1		No	268 (9.1)	22
	3	31 (1.1)	36.7	**Grade of differentiation (worst)**	Well (G1)	69 (2.4)	70.6
**ASA grade**	1	359 (12.2)	56.9		Moderate (G2)	730 (24.9)	56.4
	2	1604 (54.7)	55.9		Poor/anaplastic (G3/G4)	1674 (57.1)	50.6
	3	935 (31.9)	48.3		Unable to determine (GX)	268 (9.1)	57.4
	4	33 (1.1)	18.9		Missing	190 (6.5)	50.2
**Neoadjuvant treatment**	None	1525 (52.0)	55.3	**Adjuvant treatment**	No	2280 (77.8)	53.8
	Chemotherapy	1406 (48.0)	50.5		Yes	651 (22.2)	51.5

Data given as absolute number with percentages in parentheses.

*Major gastrointestinal resections including oesophagectomy and gastrectomy.

† Anastomotic leak defined as severe disruption to anastomosis, regardless of method of detection or intervention.

A total of 10 variables were identified as important and included in the final model. These were age, cT stage, cN stage, WHO performance status, ASA grade, pT/ypT, total number of positive lymph nodes, grade of differentiation (good, moderate, poor/anaplastic), completeness of resection (R0/R1) and neoadjuvant treatment received (*Fig. S3*).

**Fig. 2:**
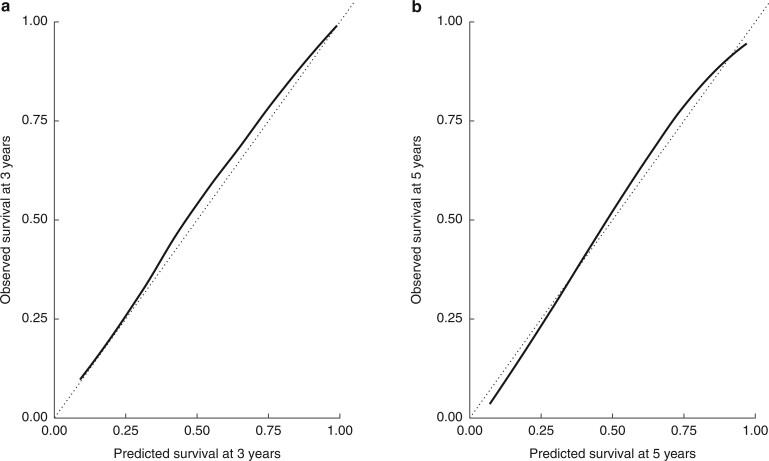
Calibration of predictions **a** 3 years and **b** 5 years after surgery. The dotted line represents the ideal and the solid line the model’s performance on internal validation

The model demonstrated excellent discrimination on internal validation, with a tAUC of 0.80 (95 per cent c.i. 0.78 to 0.82) at 5 years and a C-index of 0.76 (95 per cent c.i. 0.75 to 0.77). The tAUC using pTNM stage alone was 0.75. Agreement between predicted and observed survival was also excellent, with a wide spread of predictions observed for both 3-year (14.8–98.3 (i.q.r. 43.2–84.4) per cent) and five-year (9.4–96.1 (i.q.r. 31.7–73.8) per cent) survival (*Fig. 2*; *Fig. S4*). The integrated Brier score was 0.137 (95 per cent c.i. 0.133 to 0.140). Importantly, the discrimination of the model exceeds that achieved using TNM stage (tAUC 0.81 *versus* 0.76 *P* < 0.001). A wide range of survival estimates are also seen for each TNM stage group (*Fig. S5*).

**Fig. 3:**
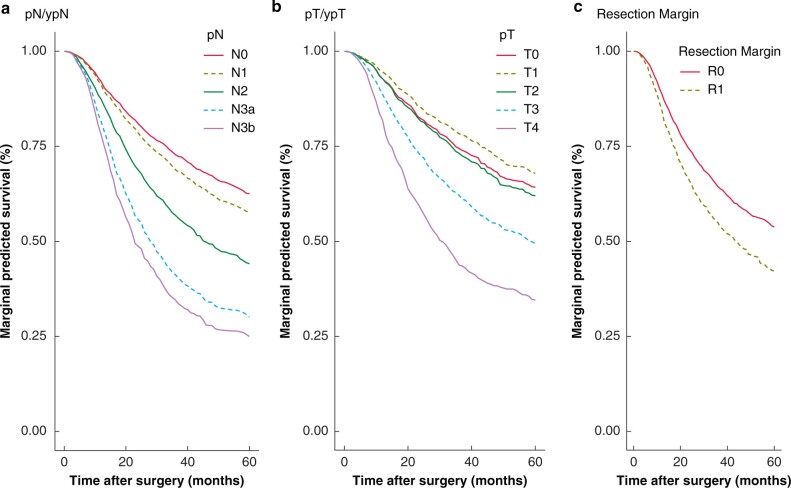
Variable effects on survival Average predicted survival is shown from 0–60 months for **a** pN stage, **b** pT stage and **c** resection margin

**Fig. 4:**
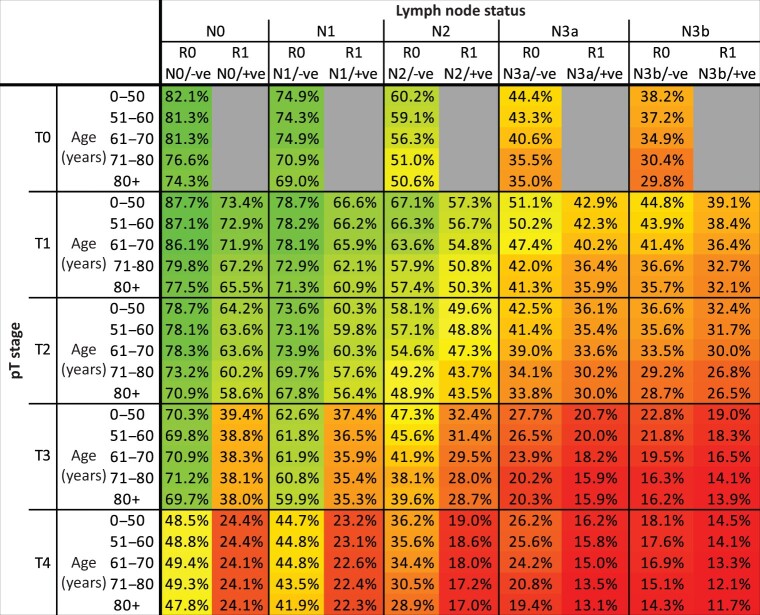
Predicted 5-year survival for combinations of selected variables Colours represent differing prognosis, with green more favourable, and red less favourable

The most important variables were number of positive lymph nodes, pT stage and completeness of resection, as visualized in survival curves shown in *Fig. 3*. The mean predicted survival (across combinations of other variables) to 5 years (the RMST) varied significantly for different characteristics, for example for pN0 the RMST was 46.4 months compared with 29.3 months for N3b patients. This corresponded to an LED of 17.1 months and an LER of 0.63. *Table S3*, illustrates the RMST, LED and LER for all variables. Although the magnitude of effect overall is small for several of the variables, in individual cases this may not be the case due to the nature of variable interactions. Advanced pN/pT (pN2/3a/3b and pT3/4) and a R1 resection exhibited an LER that clearly increases throughout the period of follow-up, indicating diverging survival trajectories and a persistent effect on prognosis for at least 4 years for pN/pT and the entirety of follow-up for R1 resection (*Fig. S6*). Notably, a Cox model trained using the same variables clearly violates the proportional hazards assumption (*P* = 0.013). One limitation of traditional estimates of importance is that variable interactions that are modelled in the RSF are ignored. To address this, *Fig. 4* gives an overview of the average predicted 5-year survival for combinations of the most important variables in addition to patient age. The importance of age can be seen to diminish with increasing tumour burden.

### Example cases

To illustrate the utility of the model, four example cases are described below.

- Case 1. A 50-year-old female patient, ASA 1, with a cT3N1 tumour, undergoes and completes neoadjuvant chemotherapy followed by a gastrectomy. Postoperative pathology reveals a pT4 well differentiated tumour with one positive lymph node and a complete resection margin (R0).
- Case 2. A 60-year-old male patient, ASA 3, with a cT3N1 tumour, undergoes but does not complete neoadjuvant chemotherapy followed by a gastrectomy. Postoperative pathology reveals a pT4 poorly differentiated tumour with three positive lymph nodes and an involved resection margin (R1).
- Case 3. A 50-year-old female patient, ASA 1, with a cT3N1 tumour, undergoes and completes neoadjuvant chemotherapy followed by a gastrectomy. Postoperative pathology reveals a pT1 well differentiated tumour with no positive lymph nodes and a complete resection margin (R0).
- Case 4. A 60-year-old male patient, ASA 3, with a cT3N1 tumour, undergoes but does not complete neoadjuvant chemotherapy followed by a gastrectomy. Postoperative pathology reveals a pT1 poorly differentiated tumour with no positive lymph nodes and a complete resection margin (R0).

**Fig. 5:**
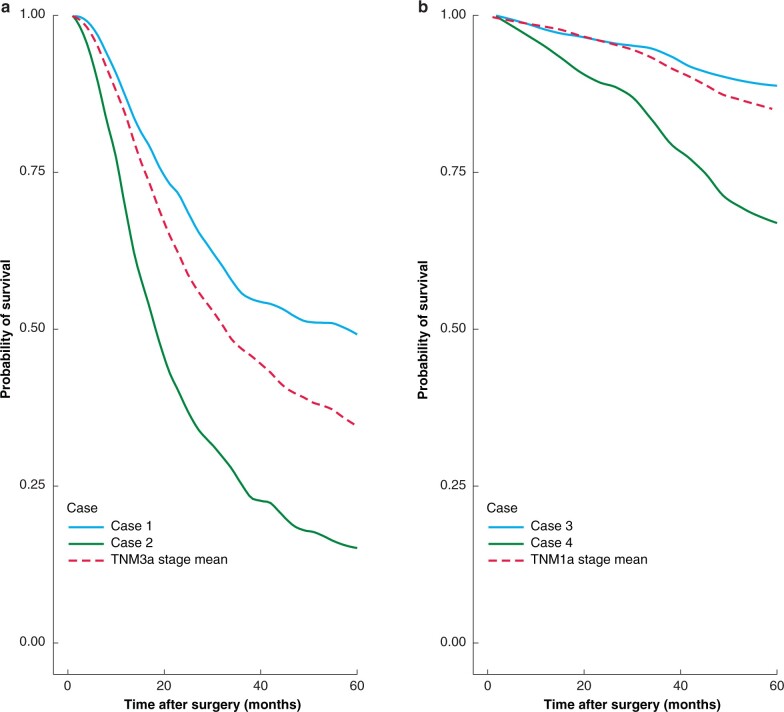
Predicted survival **a** Stage 3a and **b** stage 1a example cases and mean survival

Both cases 1 and 2 fall into the same pTNM stage group (3a), however their predicted survival trajectories show considerable differences (*Fig. 5*) with 45.7 per cent 5-year survival for case 1 and 17.0 per cent for case 2 (compared with a stage average survival of 34.4 per cent at 5 years). Similarly, cases 3 and 4 are both stage 1a, but exhibit substantial variation in 5-year survival at 88.7 and 66.5 per cent respectively.

## Discussion

This study describes the derivation and validation of a robust machine learning model for prediction of overall survival for surgically treated non-metastatic gastric adenocarcinoma. The model uses routine clinicopathological data which should be available for every case without additional investigations, to deliver predictions of survival to 5 years. The model provides accuracy in excess of traditional TNM staging to enable the delivery of personalized survival predictions, with a large spread of predictions within each TNM staging group that allows discrimination in excess of TNM staging.

Strengths of this study include the large population-based dataset used to derive the model, which is larger than those used in many previously published prognostication tools. The data are reflective of modern practice, including only patients diagnosed since 2012, with a high rate of neoadjuvant treatment (48 per cent) and D2 nodal dissection (83 per cent), with surgery performed in high-volume specialist centres. Observed overall survival exceeded recent trials, with more than one in two patients surviving to 5 years^36^^,^^37^. A machine learning non-linear approach (RSF) allowed more accuracy than otherwise could be achieved, is technically novel and has generated insight into how the importance of variables varies over time. The TRIPOD criteria for predictive modelling were also adhered to. Limitations include the retrospective nature of the study and lack of external validation cohort. An internal validation process was conducted using a bootstrap technique^38^ to assess the degree of optimism in the model’s discrimination and calibration, and its performance was maintained. External validation is still required to demonstrate its generalizability, but the importance of the T stage and nodal positive variables suggest the model is likely to be transportable to other populations. There was a moderate amount of missing data within the dataset which may introduce bias into the analysis, however this effect was minimized using multiple imputation. Lauren histological classification^39^, a well recognised and prognostic variable in gastric cancer^40^, was not available for this study and may provide additional information above differentiation grade if added in the future, although, as the diffuse type are poorly differentiated by definition, there will be extensive overlap with the classification employed here.

This model provides a broad range of survival estimates, with substantially more variability than TNM stage both overall and within each staging group, as is clearly illustrated in the example cases. The precision facilitates use of the model in several clinical settings. Firstly, more reliable information on long-term prognosis can be given to patients. Research to understand how best to relay data to patients is ongoing and this is undoubtedly an ethically complex area, particularly when the prognosis is poor. However, withholding accurate information from patients is unlikely to be prudent. Secondly, targeting follow-up and/or additional treatment to those who most require it is vital to improving outcomes and accurate prognostication with low burden of data collection (as is the case with clinicopathological models) is vital to achieve that. This is particularly important when introducing novel agents or when effect sizes appear small, as they are with current agents.

The most important variables identified (lymph node status, pT stage, resection margin) are well recognised as highly prognostic^41–43^. The demonstration of effects for these variables that persist throughout follow-up is, however, novel and informative in the context of a modelling strategy that allows for time-varying effects. In this study, only a small overall magnitude of effect of neoadjuvant treatment was identified, with no benefit seen for cases where chemotherapy was not completed. This is in contrast to the Medical Research Council Adjuvant Gastric Infusional Chemotherapy (MAGIC)^44^ and Actions Concertées dans les Cancer Colorectaux et Digestifs (ACCORD) trials^45^, which demonstrated a substantial survival benefit of neoadjuvant treatment, establishing the rationale for the widespread use of neoadjuvant chemotherapy for gastric adenocarcinoma in western countries. In reality, the effect of chemotherapy varies at an individual level, with some patients gaining a substantial benefit from the treatment (those who respond) and the majority gaining no benefit at all. The non-linear nature of the RSF which includes interactions with other variables allows response (as reflected in, for example, pT stage or the resection margin) to be accurately incorporated into prognostication, which would be challenging in a linear model and not assessed by TNM stage alone. This may also be an explanation for the counterintuitive finding of pT0 tumour having both a slightly worse observed prognosis than pT1 tumours (5-year survival 79.5 per cent pT0 *versus* 81.9 per cent pT1, *Table 1*) and life expectancy difference of -1.65 months (*Table S2*) although the number of patients with pT0 tumours was small (116 patients).

Patients in whom fewer than 15 lymph nodes were examined at resection were excluded. The purpose of this study was to derive an accurate predictive model for the most common stages of disease witnessed in clinical practice, rather than assess the prognostic importance of lymph node harvest which is of sufficient interest and complexity to warrant a separate study. After discussion the authors elected to exclude patients with an ‘inadequate’ lymph node resection because including these patients would introduce unreliable data into the model-derivation process (due to possible under-staging) and make predictions on patients who were treated as per national or international standards less reliable. The authors were also mindful that some patients with an apparent ‘inadequate’ lymph node harvest would have received a planned D1 dissection for early-stage disease. The model is not designed to be used in this patient group, rather for the classical presentation of locally advanced gastric cancer and limited to those patients with an adequate lymphadenectomy.

The variable selection method excluded variables that may have been expected to influence survival significantly, notably site of tumour and receipt of adjuvant therapy. It is reasonable to extrapolate that the difference in survival observed for different tumour sites (for example, 5-year survival 40.7 per cent for pyloric tumours compared with 56.7 per cent for Siewert III gastro-oesophageal junction tumours, *Table 1*) is largely due to differences in tumour stage at these sites, however it is surprising to see no improvement of survival with the administration of adjuvant treatment. There are several possible explanations for this, including insensitivity of the modelling approach (particularly as survival is worse after adjuvant treatment on univariable analysis, as it is more often given only to cases with more advanced disease). The margin of effect of adjuvant treatment seen in randomized trials does appear modest^46^, however, particularly in the context of a cohort in which the majority of patients underwent neoadjuvant treatment^47^.

A robust tool for prediction of overall survival after gastrectomy for adenocarcinoma has been derived using an RSF methodology. It provides accurate predictions of outcome in excess of TNM staging using routinely collected clinicopathological data. It is available at: uoscancer.shinyapps.io/AugisSurvG. Future work to validate the authors’ findings in external cohorts would be beneficial, and prospective validation before use to stratify treatment and follow-up is important.

## Supplementary Material

znab237_Supplementary_Data
