# Supplementary material for: Prediction of long-term survival after gastrectomy using random survival forests
Source: Br J Surg. 2021 Jul 16;108(11):1341–50. doi: 10.1093/bjs/znab237 (PMC10364915; doi:10.1093/bjs/znab237)
Supplement: znab237_Supplementary_Data [file znab237_supplementary_data.docx]

# Supplementary information

Supplementary Table 1 Characteristics of study cohort in comparison to patients with inadequate lymph node harvest

|  | | Lymph node Harvest | |  |  | | Lymph node Harvest | |  |
| --- | --- | --- | --- | --- | --- | --- | --- | --- | --- |
| Characteristics | | ≥15 | <15 | p§ | Characteristics | | ≥15 | <15 | p§ |
|  |  | 2931 | 754 |  |  |  | 2931 | 754 |  |
| Age | | 71 [61, 77] | 73 [64, 79] | <0.001*¶ | Annual volume of major upper gastrointestinal resections* | 1 to 30 | 245 (8.4) | 91 (12.1) | 0.002* |
| Site of Tumour | Siewert III | 416 (14.2) | 68 (9.0) | <0.001* |  | 31 to 60 | 1563 (53.3) | 365 (48.4) |  |
|  | Fundus | 195 (6.7) | 41 (5.4) |  |  | >60 | 1123 (38.3) | 298 (39.5) |  |
|  | Body | 1250 (42.6) | 308 (40.8) |  | Annual volume of major gastrectomy | 1 to 15 | 834 (28.1) | 211 (27.6) | <0.001* |
|  | Antrum | 689 (23.5) | 210 (27.9) |  |  | 16 to 30 | 1654 (55.7) | 479 (62.7) |  |
|  | Pylorus | 381 (13.0) | 127 (16.8) |  |  | 30+ | 483 (16.3) | 74 (9.7) |  |
| cT | T0/is/1 | 295 (11.5) | 141 (22.3) | <0.001* | Laparoscopic Approach | | 439 (15.0) | 151 (20.0) | 0.001* |
|  | T2 | 579 (22.5) | 195 (30.9) |  | Any Complication | | 679 (23.3) | 143 (19.0) | 0.015* |
|  | T3 | 1218 (47.4) | 231 (36.6) |  | Anastomotic Leak | | 93 (3.2) | 18 (2.4) | 0.311 |
|  | T4 | 476 (18.5) | 64 (10.1) |  | pT/ypT | T0 | 116 (4.0) | 41 (5.4) | <0.001* |
| cN | N0 | 1460 (51.8) | 486 (68.3) | <0.001* |  | T1 | 591 (20.2) | 252 (33.4) |  |
|  | N1 | 882 (31.3) | 155 (21.8) |  |  | T2 | 454 (15.5) | 128 (17.0) |  |
|  | N2 | 369 (13.1) | 57 (8.0) |  |  | T3 | 1004 (34.3) | 183 (24.3) |  |
|  | N3 | 108 (3.8) | 14 (2.0) |  |  | T4 | 766 (26.1) | 150 (19.9) |  |
| Performance Status | 0 | 1409 (48.1) | 361 (47.9) | 0.126 | R1 | | 268 (9.1) | 72 (9.5) | 0.785 |
|  | 1 | 1201 (41.0) | 288 (38.2) |  | Grade of differentiation | G1 | 69 (2.5) | 30 (4.4) | 0.002* |
|  | 2 | 288 (9.8) | 90 (11.9) |  |  | G2 | 730 (26.6) | 199 (29.4) |  |
|  | 3 | 31 (1.1) | 14 (1.9) |  |  | G3/4 | 1674 (61.1) | 368 (54.4) |  |
|  | 4 | 2 (0.1) | 1 (0.1) |  |  | GX | 268 (9.8) | 80 (11.8) |  |
| ASA | 1 | 359 (12.2) | 118 (15.6) | 0.002* | Adjuvant Treatment | | 872 (29.8) | 165 (21.9) | <0.001* |
|  | 2 | 1604 (54.7) | 369 (48.9) |  | Extent of Nodal Dissection | None | 234 (8.0) | 98 (13.0) | <0.001* |
|  | 3 | 935 (31.9) | 250 (33.2) |  |  | D0 | 21 (0.7) | 12 (1.6) |  |
|  | 4 | 33 (1.1) | 17 (2.3) |  |  | D1 | 240 (8.2) | 169 (22.4) |  |
| Female Gender | | 1017 (34.7) | 254 (33.7) | 0.633 |  | D2 | 2425 (82.7) | 474 (62.9) |  |
| Neoadjuvant Treatment | | 1406 (48.0) | 256 (34.0) | <0.001* |  | D3 | 11 (0.4) | 1 (0.1) |  |
| Data presented as absolute number (%) and median (IQR), *<0.05, § χ2 test, except ¶ Mann–Whitney U test **Major gastrointestinal resections including oesophagectomy or gastrectomy | | | | | | | | | |

Supplementary Table 2 Candidate Predictors

| Preoperative | Operative | Pathological/Post-operative |
| --- | --- | --- |
| Gender | Approach | pT/ypT |
| Age | Number of Procedures | Total number of positive lymph nodes |
| Site of Tumour | Any Complications | Grade of differentiation |
| cT | Anastomotic Leak | Completeness of resection (R0/R1) |
| cN | Cardiac Complications | Adjuvant Treatment |
| IHD | Respiratory Complications |  |
| COPD | Pleural Effusion |  |
| CKD | Pneumonia |  |
| DM | Extent of Nodal Dissection |  |
| CVD |  |  |
| PS |  |  |
| ASA |  |  |
| Hospital Volume (Major Upper GI Resection) |  |  |
| Hospital Volume (Gastrectomy Alone) |  |  |
| Neoadjuvant Treatment |  |  |

Supplementary Table 3 Predicted mean survival time according to covariate selection, restricted to five years

| Characteristic |  | Predicted mean survival time (Months) | Life Expectancy Difference (Months 95% CI) | Life Expectancy Ratio (95% CI) |
| --- | --- | --- | --- | --- |
| Age | 18-50 | 43.3 | Reference | 1 |
|  | 51-60 | 43.1 | -0.17 (-3.99 to 3.65) | 1 (0.91 to 1.08) |
|  | 61-70 | 42.7 | -0.55 (-4.37 to 3.27) | 0.99 (0.9 to 1.07) |
|  | 71-80 | 41.5 | -1.8 (-5.62 to 2.02) | 0.96 (0.87 to 1.04) |
|  | 80+ | 41.0 | -2.25 (-6.07 to 1.57) | 0.95 (0.86 to 1.03) |
| cT | T0/is/1 | 42.5 | Reference | 1 |
|  | T2 | 42.3 | -0.14 (-3.21 to 2.92) | 1 (0.92 to 1.07) |
|  | T3 | 41.8 | -0.72 (-3.78 to 2.34) | 0.98 (0.91 to 1.05) |
|  | T4 | 42.3 | -0.14 (-3.2 to 2.93) | 1 (0.92 to 1.07) |
| cN | N0 | 42.4 | Reference | 1 |
|  | N1 | 42.2 | -0.27 (-3.33 to 2.79) | 0.99 (0.92 to 1.07) |
|  | N2 | 41.4 | -0.97 (-4.02 to 2.08) | 0.98 (0.91 to 1.05) |
|  | N3 | 40.6 | -1.81 (-4.85 to 1.23) | 0.96 (0.89 to 1.03) |
| ASA | 1 | 42.3 | Reference | 1 |
|  | 2 | 42.6 | 0.25 (-2.82 to 3.32) | 1.01 (0.93 to 1.08) |
|  | 3 | 41.3 | -1.01 (-4.06 to 2.04) | 0.98 (0.9 to 1.05) |
| Performance Status | 0 | 42.9 | Reference | 1 |
|  | 1 | 41.8 | -1.1 (-4.17 to 1.97) | 0.97 (0.9 to 1.05) |
|  | 2 | 40.3 | -2.59 (-5.64 to 0.46) | 0.94 (0.87 to 1.01) |
|  | 3 | 40.6 | -2.25 (-5.31 to 0.8) | 0.95 (0.88 to 1.02) |
| Neoadjuvant Treatment | None | 42.2 | Reference | 1 |
|  | Chemotherapy-Completed | 42.0 | -0.23 (-3.28 to 2.83) | 0.99 (0.92 to 1.07) |
|  | Chemotherapy-not completed |  |  |  |
| Completeness of Resection | R0 | 42.6 | Reference | 1 |
|  | R1 | 37.9 | -4.71 (-7.71 to -1.7) | 0.89 (0.82 to 0.96) |
| pT Stage | T0 | 47.2 | Reference | 1 |
|  | T1 | 48.8 | 1.69 (-1.55 to 4.93) | 1.04 (0.97 to 1.11) |
|  | T2 | 46.5 | -0.66 (-3.85 to 2.54) | 0.99 (0.92 to 1.05) |
|  | T3 | 41.4 | -5.8 (-8.91 to -2.68) | 0.88 (0.82 to 0.94) |
|  | T4 | 33.9 | -13.25 (-16.29 to -10.2) | 0.72 (0.66 to 0.77) |
| pN Stage | N0 | 46.4 | Reference | 1 |
|  | N1 | 44.4 | -1.96 (-5.78 to 1.86) | 0.96 (0.88 to 1.04) |
|  | N2 | 39.1 | -7.26 (-11.08 to -3.44) | 0.84 (0.77 to 0.92) |
|  | N3a | 32.6 | -13.8 (-17.62 to -9.98) | 0.7 (0.63 to 0.77) |
|  | N3b | 29.3 | -17.09 (-20.91 to -13.27) | 0.63 (0.56 to 0.7) |
| Grade of Differentiation | G1 | 42.7 | Reference | 1 |
|  | G2 | 42.5 | -0.19 (-3.26 to 2.87) | 1 (0.92 to 1.07) |
|  | G3/4 | 42.0 | -0.71 (-3.77 to 2.35) | 0.98 (0.91 to 1.05) |
|  | GX | 42.5 | -0.17 (-3.24 to 2.89) | 1 (0.92 to 1.07) |
| All values restricted to five years. The Life expectancy difference (LED) is the change in mean survival (months) from the reference value, and the Life expectancy ratio (LER) is the corresponding ratio of this. | | | | |

Supplementary Figure 1 Kaplan–Meier estimation of survival, stratified by lymph node yield
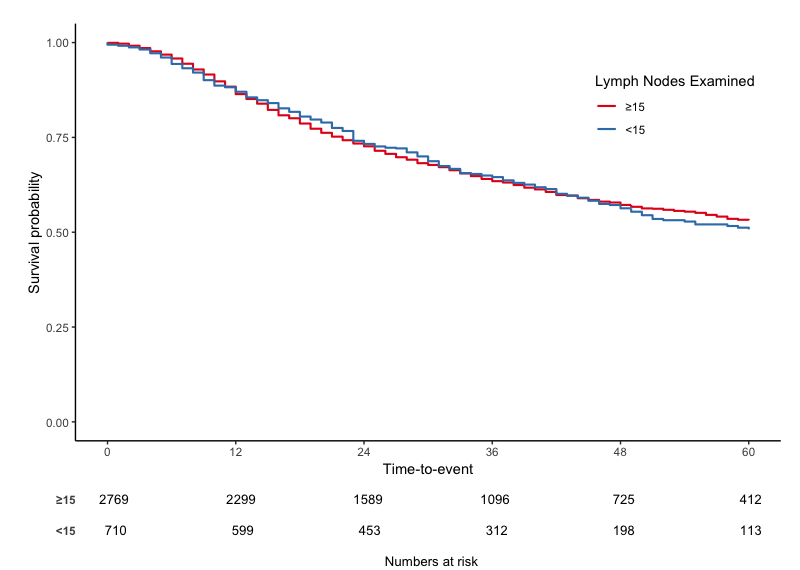


Supplementary Figure 2 Study Flow Diagram


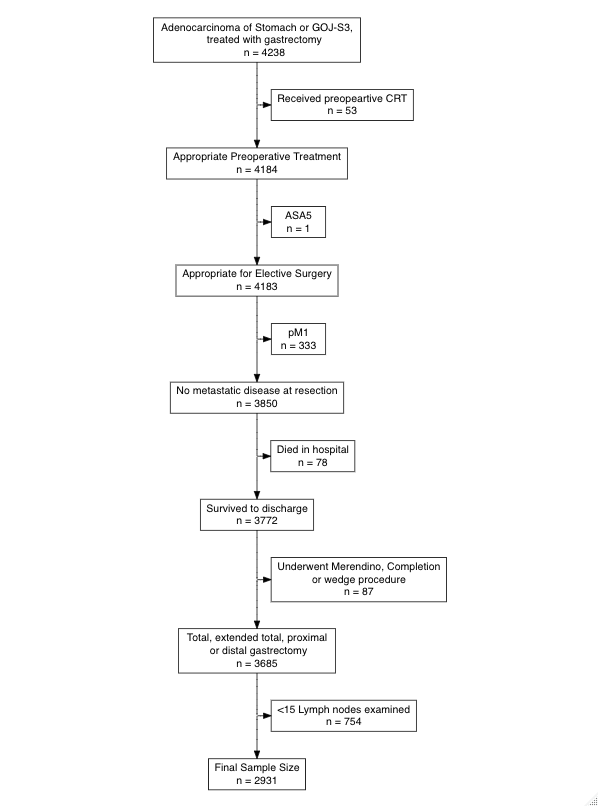


Supplementary Figure 3 Boruta variable importance. Candidate variables are compared to a set of variables that have been randomised in a Random Forest model and those with an importance to survival significantly higher than all randomised (shadow) variables are selected for inclusion.


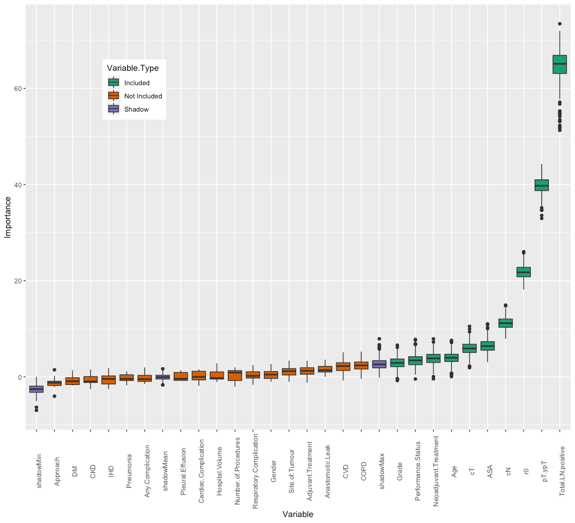


Supplementary Figure 4 Agreement between observed and predicted survival, grouped into quintiles by predicted survival at five-years post-surgery


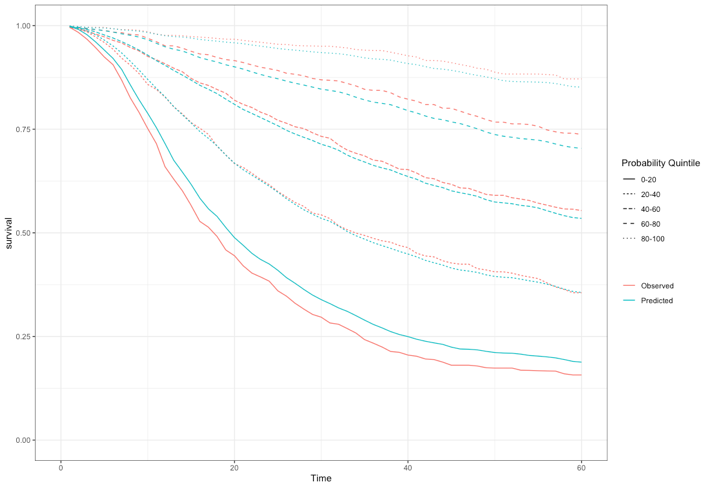


Supplementary Figure 5 Interquartile range of predictions within pTNM staging groups


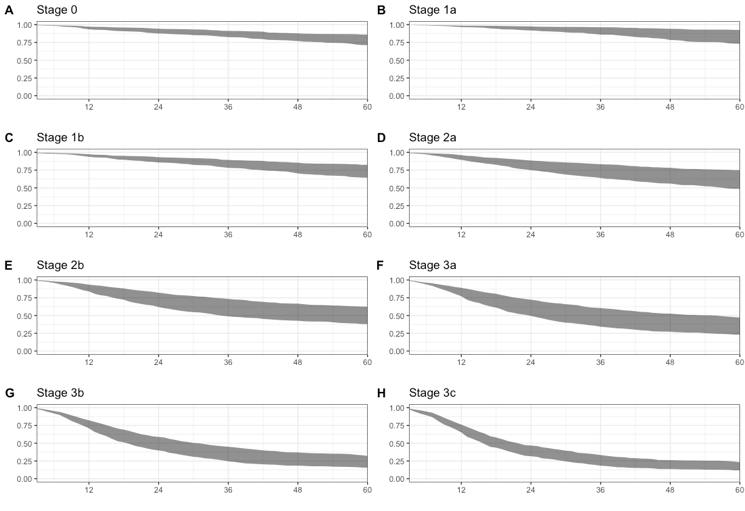


Supplementary Figure 6 Life expectancy ratio (LER) over time. Persistent effects are seen for positive resection margin. pT/pN effects plateau at about 4 years


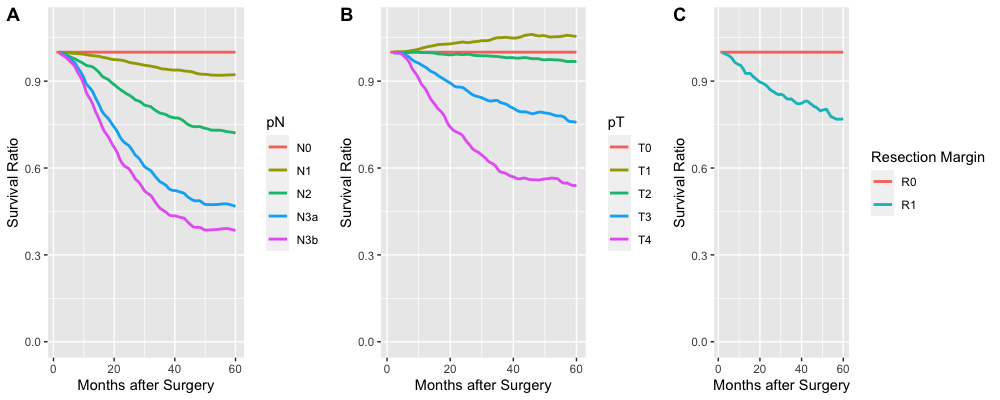


## External Validation Instructions

A basic knowledge of R is required to conduct the external validation. As the model does not generate coefficients, access to the model itself is required.

First, download the file packet from the web application in the *‘Model Details’* tab. This contains the models themselves and the manner in which dummy coding was conducted.

An example blank dataframe is also included showing the structure in which data must be presented to the model. Care should be taken to match the variables/names/factor-levels in this file. If the model fails to generate predictions, it is probably due to a discrepancy here.

Then access and download the R script from github:

<https://github.com/saqibrahmanUGI/AUGIS-Surv>

Running this script will firstly install and load the needed R packages, then batch generate predictions, calculate the tAUC and C-index, plot annual calibration curves and plot quintiles of prediction against observed KM estimates.
